# Association between social phobia and allergic asthma in adolescents

**DOI:** 10.3389/falgy.2025.1698470

**Published:** 2025-12-05

**Authors:** Shuang Han, Tao Wang, Jiaojiao Wang, Zhihua Han, Pengfei Wang

**Affiliations:** 1 Department of Pulmonary and Critical Care Medicine, Shandong Provincial Key Medical and Health Discipline, Qingdao Central Hospital, University of Health and Rehabilitation Sciences, Qingdao, Shandong, China; 2 Department of General Surgery, North Campus of Qingdao Haici Traditional Chinese Medicine Medical Group, Qingdao Hongdao People’s Hospital, Qingdao, Shandong, China; 3 Department of Medical Record Management, Shandong Provincial Key Medical and Health Discipline, Qingdao Central Hospital, University of Health and Rehabilitation Sciences, Qingdao, Shandong, China; 4 Medical Insurance Office, North Campus of Qingdao Haici Traditional Chinese Medicine Medical Group, Qingdao Hongdao People’s Hospital, Qingdao, Shandong, China

**Keywords:** adolescents, asthma, inflammation, social phobia, pulmonary function

## Abstract

**Background:**

Social phobia and asthma pose threats to the health of adolescents at the psychological and physical levels, respectively. The aim of this study was to explore the association between social phobia and asthma in this population.

**Methods:**

A total of 337 adolescent asthma patients and 337 adolescent controls were included. Social phobia status was assessed using the Mini Social Phobia Inventory (Mini-SPIN) and the Social Anxiety Scale for Children (SASC). The ratio of forced expiratory volume in 1 s to forced vital capacity (FEV1/FVC), the percentage of forced expiratory volume in 1 s to its predicted value (FEV1%pred), peak expiratory flow, and peripheral levels of plasma eosinophil, immunoglobulin E (IgE), leukotriene, and histamine were also measured. Multivariate logistic or linear regression analyses were used to evaluate the associations between social phobia-related variables and asthma-related variables.

**Results:**

Elevated scores on the Mini-SPIN and SASC scales were associated with an increased risk of asthma in adolescents (both *P* < 0.001). This association remained consistent among adolescents with new-onset asthma (both *P* < 0.001) and those experiencing asthma recurrence in adolescence following a childhood asthma history (both *P* < 0.001). Meanwhile, higher scores on both scales correlated with decreased FEV1/FVC (both *P* < 0.001) and FEV1%pred (*P* = 0.001 and *P* = 0.002, respectively) and elevated leukotriene levels (*P* < 0.001 and *P* = 0.001, respectively). However, neither scale showed an association with plasma eosinophil, IgE, or histamine levels.

**Conclusion:**

Among adolescents, there was a significant association between social phobia and asthma.

## 1 Introduction

Currently, adolescent asthma (aged 10–19 years) has increasingly emerged as a major public health concern (1). According to epidemiological data, the prevalence of this type of disease has been increasing in recent years. In some developed countries, the prevalence can even reach 10%–20% (2, 3). Adolescent asthma not only significantly affects daily life and academic performance of adolescents but, due to long-term repeated attacks, may also cause irreversible damage to lung function and even endanger life (4, 5).

Anxiety and asthma may be closely linked. On one hand, asthma significantly disrupts daily lives of patients, increasing psychological stress and triggering anxiety, and multiple studies have confirmed that asthma patients are more prone to anxiety than non-asthmatic controls (6, 7). On the other hand, at the theoretical level, long-term anxiety may induce abnormalities in the nervous, endocrine, and immune systems, thereby enhancing airway reactivity and increasing the frequency and severity of asthma exacerbations. Although no direct studies have validated this mechanism to date, research has shown that children born to anxious pregnant women have a higher risk of developing asthma than those born to non-anxious pregnant women (8, 9), which indirectly suggests that anxiety may contribute to the onset and progression of asthma.

Among anxiety disorders, social phobia warrants special attention in adolescents due to its unique clinical characteristics and high prevalence in this age group (10). Briefly, social phobia is triggered by highly specific situations. Intense fear and avoidance responses are elicited only when an individual is in social settings. This characteristic makes social phobia more covert than general anxiety and easily overlooked in daily life. Notably, social phobia is more prevalent among adolescents (11). Adolescents in this age group are in a crucial period of frequent social activities and gradual psychological maturation, and the impact of social phobia on their lives, studies, and mental health cannot be underestimated. Therefore, exploring the association between social phobia and asthma in this specific age group is of great practical significance. On one hand, it helps identify high-risk asthma patients potentially affected by social phobia, enabling the development of personalized treatment plans that include psychological interventions. On the other hand, understanding the relationship between social phobia and asthma in adolescents can provide new ideas for the early prevention and intervention of social phobia in this group. However, research focusing on the association between social phobia and asthma, specifically in adolescents, remains in its infancy.

Therefore, this study aims to employ a case–control design, with adolescents aged 10–19 years as the research participants, to explore the association between social phobia and asthma in this specific population. On the scientific front, this study helps fill the gap in this field and lays a foundation for future investigations into comorbidity mechanisms. Clinically, its findings may facilitate bidirectional health management and improved outcomes for individuals with “asthma–social phobia” comorbidity.

## 2 Materials and methods

### 2.1 Ethical requirements

This study was approved by the Medical Ethics Committee of Qingdao Central Hospital and was conducted in accordance with the requirements of the Declaration of Helsinki of the World Medical Association. All participants or their guardians consented to participate in this study and signed the informed consent forms.

### 2.2 Participants

A total of 337 adolescents with asthma were enrolled from 24 communities in Qingdao City, China, between 1 January and 31 December 2023. Of the 348 subjects who met the eligibility criteria, 11 declined participation, resulting in a final sample of 337 (participation rate: 96.8%) in the asthma group.

The inclusion criteria are as follows: (1) the age ranges between 10 and 19 years. (2) The medical records confirmed that the participants meet the asthma diagnostic criteria established by the Global Initiative for Asthma (GINA) 2021. The diagnostic criteria for asthma are as follows (12, 13): the presence of typical symptoms and signs of asthma; a positive bronchodilator test, a positive bronchial provocation test, or a diurnal variation rate or circadian fluctuation rate of peak expiratory flow (PEF) ≥20%, with at least one of the above three pulmonary function test results being positive; and exclusion of other diseases. (3) There has been an acute asthma attack within the most recent 3 months. The definition of an acute asthma attack is as follows (12, 13): symptoms such as wheezing, shortness of breath, and chest tightness occur suddenly, or existing symptoms worsen sharply, often accompanied by dyspnea, and characterized by decreased expiratory flow rate; the severity varies, and exacerbations may develop within several hours or days. (4) The participants have never been diagnosed with any mental or psychological diseases and have never received any related treatment. (5) They have no congenital diseases and no other serious conditions (such as type 1 diabetes mellitus, hyperthyroidism, and epilepsy).

During the same period, a 1:1 frequency-matching strategy was adopted: for each asthmatic case, a healthy control was selected from 10 neighboring households (sorted by building, floor, and apartment number) in the same community, prioritizing comparable age, sex, and ethnicity. A total of 337 controls were enrolled, forming the control group.

The inclusion criteria for the control group are as follows: (1) age between 10 and 19 years; (2) no history of asthma, congenital diseases, or other relatively serious diseases; and (3) no previous diagnosis of any mental or psychological disorders.

### 2.3 Asthma information collection

Researchers visited the households of participants in both groups to collect asthma-related information. They reviewed medical records and obtained the asthma severity classification based on the GINA 2021 guidelines (i.e., mild, moderate, severe, and very severe), the age at initial onset, and the current anti-asthmatic medications being used (Table 1). They also recorded current asthma risk factors for participants in both groups, including a history of allergies, exposure to various types of pollution, passive smoking history, personal exercise history, medical history, and family history (Table 2) (14–17). The definitions of these risk factors are provided in Supplementary Table S1.

**Table 1:** Demographic and asthma information of the participants.

Variable	Asthma (*n* = 337)	Control (*n* = 337)	*t*/*χ*^2^	*P*
Demographic information				
Male (*n*)	151 (44.8)	170 (50.4)	2.147	0.143
Age (years)	14.7 ± 2.6	14.5 ± 2.6	0.758	0.449
Han ethnicity (*n*)	313 (92.9)	306 (90.8)	0.970	0.325
GINA severity c				
Mild (*n*)	229 (68.0)	—	—	—
Moderate (*n*)	80 (23.7)	—	—	—
Severe to very severe (*n*)	28 (8.3)	—	—	—
Initial onset				
In childhood (*n*)	227 (67.4)	—	—	—
In adolescence (*n*)	110 (32.6)	—	—	—
Asthma indicator				
FEV1/FVC (%)	77.5 ± 4.2	90.2 ± 2.9	56.712	<0.001
FEV1%pred (%)	79.5 ± 16.1	99.9 ± 2.9	22.918	<0.001
PEF (L/min)	78.8 ± 9.2	99.8 ± 1.7	41.152	<0.001
Plasma eosinophil (×10^9^/L)	0.54 ± 0.26	0.27 ± 0.13	16.780	<0.001
Plasma IgE (IU/mL)	85.7 ± 39.1	49.4 ± 25.5	14.264	<0.001
Plasma leukotriene (nmol/L)	0.51 ± 0.18	0.30 ± 0.11	18.023	<0.001
Plasma histamine (μmol/L)	0.40 ± 0.12	0.26 ± 0.08	18.343	<0.001
Anti-asthmatic medication				
Inhaled *β*-receptor agonists (*n*)	279 (82.8)	—	—	—
Inhaled anticholinergics (*n*)	128 (38.0)	—	—	—
Inhaled glucocorticoids (*n*)	240 (71.2)	—	—	—
Oral leukotriene modifiers (*n*)	148 (43.9)	—	—	—

GINA, Global Initiative for Asthma; FEV1/FVC, ratio of forced expiratory volume in 1  to forced vital capacity; FEV1%pred, percentage of forced expiratory volume in 1 s to its predicted value; PEF, peak expiratory flow.

Continuous variables are expressed as mean ± standard deviation. The differences between groups are evaluated using independent-sample *t*-tests, and the *t*-value and *P*-value are reported. Categorical variables are presented as frequencies (proportions). The differences between groups are evaluated using chi-square tests, and the χ^2^-value and *P*-value are reported. A *P*-value <0.05 indicates statistical significance.

**Table 2:** Risk factors for asthma in the participants.

Variable	Asthma (*n* = 337)	Control (*n* = 337)	*t*/*χ*^2^	*P*
Food allergy (*n*)	65 (19.3)	44 (13.1)	4.826	0.028
Inhalant allergy (*n*)	187 (55.5)	113 (33.5)	32.895	<0.001
Industrial pollution (*n*)	105 (31.2)	73 (21.7)	7.817	0.005
Exhaust gas pollution (*n*)	125 (37.1)	89 (26.4)	8.873	0.003
Kitchen fume pollution (*n*)	114 (33.8)	77 (22.8)	10.002	0.002
Decoration pollution (*n*)	93 (27.6)	67 (19.9)	5.540	0.019
Passive smoking (*n*)	125 (37.1)	96 (28.5)	5.662	0.017
Lack of exercise (*n*)	121 (35.9)	94 (27.9)	4.979	0.026
Obesity (*n*)	107 (31.8)	63 (18.7)	15.230	<0.001
Respiratory tract infection (*n*)	161 (47.8)	74 (22.0)	49.450	<0.001
Family history of asthma (*n*)	76 (22.6)	25 (7.4)	30.292	<0.001

Continuous variables are expressed as mean ± standard deviation. The differences between groups are evaluated using independent-sample *t*-tests, and the *t*-value and *P*-value are reported. Categorical variables are presented as frequencies (proportions). The differences between groups are evaluated using chi-square tests, and the *χ*^2^-value and *P*-value are reported. A *P*-value <0.05 indicates statistical significance.

The participants in both groups were invited to a designated local community hospital, where three indicators were measured using a Lepu S600 (Lepu Diagnostic Technology Co., Ltd., Beijing, China) pulmonary function tester: the ratio of forced expiratory volume in 1 s to forced vital capacity (FEV1/FVC), the percentage of forced expiratory volume in 1 s to its predicted value (FEV1%pred), and PEF (Table 1). The predicted value of FEV1 was calculated using the race-neutral Global Lung Function Initiative (GLI) 2022 reference equations, which require only age, height, and sex as predictive factors to avoid interpretation bias caused by racial stratification (18). Three milliliters of fasting venous blood were collected, and the levels of plasma eosinophil, immunoglobulin E (IgE), leukotriene, and histamine were measured using a hematology analyzer, immunoturbidimetry, enzyme-linked immunosorbent assay (ELISA), and fluorescence method, respectively (Table 1). All pulmonary function and peripheral indicator measurements were performed by professional physicians in this community hospital, and quality control was the responsibility of the quality control department of this hospital.

### 2.4 Social phobia assessment

During household visits, researchers assessed the social phobia status of participants in both groups using the Mini Social Phobia Inventory (Mini-SPIN) and the Social Anxiety Scale for Children (SASC). In addition, the Beck Anxiety Inventory (BAI) was employed to evaluate the general anxiety status of participants in both groups (Table 3). Since social phobia is a subtype of anxiety disorder and is often comorbid with generalized anxiety, this study adopted the aforementioned combination of scales to ensure the most comprehensive assessment of the social phobia and anxiety status of participants.

**Table 3:** Social phobia status of the participants.

Variable	Asthma (*n* = 337)	Control (*n* = 337)	*t*/*χ*^2^	*P*
Social phobia scores				
Mini-SPIN	3.8 ± 2.3	3.1 ± 1.7	4.732	<0.001
SASC	37.3 ± 8.0	34.9 ± 5.9	4.491	<0.001
BAI	6.8 ± 5.5	4.8 ± 3.7	5.503	<0.001
Social phobia symptoms				
Specific scene avoidance (*n*)	71 (21.1)	49 (14.5)	4.907	0.027
Daily social avoidance (*n*)	54 (16.0)	32 (9.5)	6.451	0.011
Emotional anxiety (*n*)	117 (34.7)	92 (27.3)	4.335	0.037
Physical reactions (*n*)	77 (22.8)	60 (17.8)	2.648	0.104
Excessive self-focus (*n*)	75 (22.3)	51 (15.1)	5.623	0.018
Negative self-assessment (*n*)	92 (27.3)	68 (20.2)	4.721	0.030
Sensitivity to others' evaluations (*n*)	79 (23.4)	55 (16.3)	5.365	0.021
Duration of core symptoms				
Specific scene avoidance (years)	2.0 ± 1.3	1.5 ± 1.0	2.285	0.024
Daily social avoidance (years)	2.1 ± 1.2	1.7 ± 1.0	1.859	0.067
Emotional anxiety (years)	2.0 ± 1.2	1.6 ± 1.0	2.838	0.005

Mini-SPIN, Mini Social Phobia Inventory; SASC, Social Anxiety Scale for Children; BAI, Beck Anxiety Inventory.

Continuous variables are expressed as mean ± standard deviation. The differences between groups are evaluated using independent-sample *t*-tests, and the *t*-value and *P*-value are reported. Categorical variables are presented as frequencies (proportions). The differences between groups are evaluated using chi-square tests, and the *χ*^2^-value and *P*-value are reported. A *P*-value <0.05 indicates statistical significance.

The Mini-SPIN consists of three items and uses a scoring standard ranging from 0 to 4. It scores the levels of fear, avoidance, and distress in social situations. Its theoretical score ranges from 0 to 12, with higher scores indicating more severe social phobia (19).

The SASC contains 22 items and adopts a three-level scoring standard. It evaluates three dimensions, namely, fear of negative evaluation, social avoidance and distress, and fear of new situations. Its theoretical score ranges from 22 to 66, with higher scores indicating more severe social phobia (20).

The BAI has 21 items and uses a four-level scoring system. Its theoretical score ranges from 0 to 63, with higher scores indicating more severe anxiety (21).

Researchers recorded the social phobia symptoms of participants and the duration of core symptoms in both groups (Table 3). They also documented the risk factors related to social phobia, which mainly included family factors, school-related factors, personal factors, and external factors (Table 4). The definitions of these risk factors are provided in Supplementary Table S1.

**Table 4:** Risk factors for social phobia in the participants.

Variable	Asthma (*n* = 337)	Control (*n* = 337)	*t*/*χ*^2^	*P*
Family factor				
Relative's death (*n*)	19 (5.6)	16 (4.7)	0.271	0.603
Parental divorce (*n*)	42 (12.5)	31 (9.2)	1.859	0.173
Parent unemployed (*n*)	61 (18.1)	51 (15.1)	1.071	0.301
Overindulgence (*n*)	74 (22.0)	65 (19.3)	0.734	0.392
Lack of parental care (*n*)	52 (15.4)	44 (13.1)	0.777	0.378
School-related factor				
Poor academics (*n*)	55 (16.3)	48 (14.2)	0.562	0.454
School relationship problems (*n*)	52 (15.4)	34 (10.1)	4.318	0.038
Personal factor				
Introverted and sensitive (*n*)	90 (26.7)	77 (22.8)	1.345	0.246
Sick body (*n*)	337 (100.0)	52 (15.4)	—	—
Insufficient sleep (*n*)	81 (24.0)	60 (17.8)	3.955	0.047
External factor				
Addiction to the Internet (*n*)	59 (17.5)	50 (14.8)	0.886	0.346
Lack of hobbies (*n*)	52 (15.4)	42 (12.5)	1.236	0.266
Media influence (*n*)	49 (14.5)	44 (13.1)	0.312	0.577

Continuous variables are expressed as mean ± standard deviation. The differences between groups are evaluated using independent-sample *t*-tests, and the *t*-value and *P*-value are reported. Categorical variables are presented as frequencies (proportions). The differences between groups are evaluated using chi-square tests, and the *χ*^2^-value and *P*-value are reported. A *P*-value <0.05 indicates statistical significance.

Although the aforementioned scales are self-report instruments, some participants were relatively young adolescents (e.g., 10–14 years old), which may compromise the reliability of self-reported results; therefore, uniformly trained researchers administered the assessments in accordance with the standardized procedures of each scale. To minimize subjective bias, a “dual-researcher simultaneous independent scoring” strategy was adopted: two researchers independently scored social phobia symptoms of the same participant based on the scale criteria, without influencing one another. After the completion of all assessments, the kappa test was used to analyze the consistency of the scoring results between the two researchers (22). The results showed a kappa value of 0.85 [95% confidence interval (95% CI): 0.78–0.92], with *P*-value <0.001. According to kappa consistency criteria, a value ≥0.80 indicates strong inter-rater agreement. Combined with the statistically significant result, this confirms that the assessment process was sufficiently objective and reliable, significantly mitigating the impact of individual assessor bias on data quality.

### 2.5 Statistical analysis

Continuous variables were expressed as mean ± standard deviation. The differences between groups were evaluated using an independent samples *t*-test, and the *t*-value and *P*-value were reported. Categorical variables were presented as frequencies and constituent ratios. The differences between groups were assessed using the chi-square test, and the *χ*^2^-value and *P*-value were reported.

The independent association between the three aforementioned scores (two social phobia scores and one anxiety score) and asthma (a categorical variable) was evaluated using multivariate logistic regression, with odds ratios (ORs), 95% CIs, and *P*-values reported (Table 5). In addition, the independent associations between these three scores and asthma indicators (continuous variables) were assessed using multivariate linear regression, with beta coefficients, 95% CIs, and *P*-values reported (Table 6). During this process, potential confounders were adjusted for, including demographic characteristics (gender, age, Han ethnicity; Table 1) and risk factors for social phobia and asthma (all listed in Tables 2, 4).

**Table 5:** Multivariate logistic regression analysis of the correlation between social phobia and asthma.

Variable	Multivariate model		
	*P*	OR	95% CI
All subjects			
Mini-SPIN vs. asthma	<0.001	1.192	1.106–1.285
SASC vs. asthma	<0.001	1.050	1.027–1.073
BAI vs. asthma	<0.001	1.097	1.060–1.136
Initial onset in childhood			
Mini-SPIN vs. asthma	<0.001	1.185	1.089–1.290
SASC vs. asthma	<0.001	1.049	1.023–1.075
BAI vs. asthma	<0.001	1.093	1.052–1.136
Initial onset in adolescence			
Mini-SPIN vs. asthma	<0.001	1.269	1.129–1.426
SASC vs. asthma	<0.001	1.067	1.031–1.104
BAI vs. asthma	<0.001	1.120	1.068–1.174

OR, odds ratio; 95% CI, 95% confidence interval; Mini-SPIN, Mini Social Phobia Inventory; SASC, Social Anxiety Scale for Children; BAI, Beck Anxiety Inventory.

The correlation between social phobia and asthma is evaluated using multivariate logistic regression, and the OR-value, 95% CI, and *P*-value are reported. During this process, the demographic information of the subjects and the risk factors for social phobia and asthma are adjusted. A *P*-value <0.05 indicates statistical significance.

**Table 6:** Multivariate linear regression analysis of the correlation between social phobia scores and asthma indicators.

Variable	Multivariate model		
	*P*	Beta	95% CI
All subjects			
Mini-SPIN vs. FEV1/FVC	<0.001	−0.533	−0.794 to −0.272
SASC vs. FEV1/FVC	<0.001	−0.140	−0.216 to −0.064
BAI vs. FEV1/FVC	<0.001	−0.267	−0.383 to −0.151
Mini-SPIN vs. FEV1%pred	0.001	−0.941	−1.494 to −0.388
SASC vs. FEV1%pred	0.002	−0.259	−0.420 to −0.098
BAI vs. FEV1%pred	<0.001	−0.456	−0.699 to −0.213
Mini-SPIN vs. eosinophil	0.079	0.008	−0.002 to 0.018
SASC vs. eosinophil	0.113	0.002	−0.001 to 0.004
BAI vs. eosinophil	0.037	0.004	0.001 to 0.008
Mini-SPIN vs. IgE	0.236	0.823	−0.537 to 2.183
SASC vs. IgE	0.265	0.226	−0.170 to 0.622
BAI vs. IgE	0.093	0.514	−0.086 to 1.114
Mini-SPIN vs. leukotriene	<0.001	0.012	0.006 to 0.018
SASC vs. leukotriene	0.001	0.003	0.001 to 0.005
BAI vs. leukotriene	<0.001	0.006	0.004 to 0.008
Mini-SPIN vs. histamine	0.119	0.004	−0.002 to 0.008
SASC vs. histamine	0.102	0.001	−0.001 to 0.003
BAI vs. histamine	0.076	0.002	−0.001 to 0.004

95% CI, 95% confidence interval; Mini-SPIN, Mini Social Phobia Inventory; SASC, Social Anxiety Scale for Children; BAI, Beck Anxiety Inventory; FEV1/FVC, ratio of forced expiratory volume in 1 s to forced vital capacity; FEV1%pred, percentage of forced expiratory volume in 1 s to its predicted value.

The correlation between social phobia score and asthma indicator is evaluated using multivariate linear regression, and the beta-value, 95% CI, and *P*-value are reported. During this process, the demographic information of the subjects and the risk factors for social phobia and asthma are adjusted. A *P*-value <0.05 indicates statistical significance.

A *P*-value <0.05 indicated statistical significance (23). All analyses were conducted using SPSS 27.0 software.

In addition, GPower 3.1.9.7 software was used to conduct *post-hoc* sample size validation, aiming to determine whether the current sample size meets the preset statistical power criteria (24).

## 3 Results

As shown in Table 1, there were no significant differences in gender, age, or ethnicity between the two groups (all *P*-values > 0.05). Compared with the control group, the levels of FEV1/FVC, FEV1%pred, and PEF in the asthma group were significantly lower (all *P*-values < 0.001), while the levels of plasma eosinophil, IgE, leukotriene, and histamine were significantly higher (all *P*-values < 0.001). In the asthma group, the majority of the patients had mild asthma, accounting for 68.0%. A total of 227 patients had disease onset during childhood with recurrence in adolescence, while the remaining 110 patients experienced their first onset during adolescence. The proportion of patients in the asthma group receiving treatment with inhaled β-receptor agonists, inhaled anticholinergics, inhaled glucocorticoids, and oral leukotriene modifiers ranged from 38.0% to 82.8%.

As shown in Table 2, asthma risk factors such as food allergy, inhalant allergy, industrial pollution, exhaust gas pollution, kitchen fume pollution, decoration pollution, passive smoking, lack of exercise, obesity, respiratory tract infection, and family history of asthma were more common in the asthma group than in the control group (*P* = 0.028, *P* < 0.001, *P* = 0.005, *P* = 0.003, *P* = 0.002, *P* = 0.019, *P* = 0.017, *P* = 0.026, *P* < 0.001, *P* < 0.001, *P* < 0.001, respectively).

As shown in Table 3, the scores on the three aforementioned scales (Mini-SPIN, SASC, and BAI) were higher in the asthma group than in the control group (all *P*-values < 0.001). Social phobia symptoms such as specific situation avoidance, daily social avoidance, emotional anxiety, excessive self-focus, negative self-assessment, and sensitivity to others' evaluations were more common among adolescents in the asthma group than among those in the control group (*P* = 0.027, *P* = 0.011, *P* = 0.037, *P* = 0.018, *P* = 0.030, *P* = 0.021, respectively). The duration of core symptoms, particularly specific situation avoidance and emotional anxiety, was longer in the asthma group than in the control group (*P* = 0.024, *P* = 0.005, respectively).

As shown in Table 4, by definition, all patients in the asthma group had the social phobia risk factor of “sick body” (attributed to their asthma diagnosis). In the control group, 15.4% of subjects had this risk factor. In addition, only two social phobia risk factors of “school relationship problems” and “insufficient sleep” were more common in the asthma group than in the control group (*P* = 0.038, *P* = 0.047, respectively). There were no significant differences in the distribution of the remaining risk factors between the two groups (all *P*-values > 0.05).

As shown in Table 5, multivariate logistic regression analysis revealed that higher Mini-SPIN, SASC, and BAI scores were associated with an increased risk of asthma (all *P*-values < 0.001). According to the age at initial onset of asthma, this study divided asthma patients into two subgroups and repeated the analyses for each subgroup. In asthma patients with the initial onset during childhood, higher Mini-SPIN, SASC, and BAI scores were similarly associated with a higher risk of asthma compared with the controls (all *P*-values < 0.001). In asthma patients with the initial onset during adolescence, higher Mini-SPIN, SASC, and BAI scores were also associated with a higher risk of asthma compared with the controls (all *P*-values < 0.001).

As shown in Table 6, multivariate linear regression analysis revealed that higher Mini-SPIN, SASC, and BAI scores were associated with lower FEV1/FVC levels (all *P*-values < 0.001) and FEV1%pred (*P* = 0.001, *P* = 0.002, *P* < 0.001, respectively). Higher Mini-SPIN, SASC, and BAI scores were associated with elevated leukotriene levels (*P* < 0.001, *P* = 0.001, *P* < 0.001, respectively). Notably, only elevated BAI scores were associated with increased eosinophil levels (*P* = 0.037), while Mini-SPIN and SASC scores showed no such association (*P* = 0.079, *P* = 0.113, respectively). Higher Mini-SPIN, SASC, and BAI scores were not associated with IgE and histamine levels (all *P*-values > 0.05).

## 4 Discussion

Exploring the association between social phobia and asthma in adolescents holds significant importance. During this critical period of physical and psychological development, social phobia and asthma pose threats to the mental and physical health of adolescents, respectively. Clarifying their association not only provides a new perspective for the integrated physical–mental assessment of adolescent health but also lays a foundation for formulating targeted health strategies.

This study employed a case–control design, enrolling 337 adolescent asthma patients and 337 adolescent controls. The findings reveal a significant association between elevated social phobia scores (assessed via the Mini-SPIN and SASC) and an increased risk of asthma. Further subgroup analyses stratified by the age at initial onset of asthma showed that this association—between higher social phobia scores and elevated asthma risk—remained consistent regardless of whether asthma first occurred in childhood (with recurrence during adolescence) or in adolescence (first onset). Since a series of demographic data and risk factors related to social phobia and asthma were adjusted for, the results obtained exhibit a certain degree of independence. To our knowledge, this is the first study conducted worldwide focusing on this specific research area.

This study did not further explore the association between asthma of different GINA severity classifications and social phobia because the sample size of patients with severe to very severe asthma was relatively small (*n* = 28, 8.3%), resulting in limited statistical power. However, the study confirmed that two key pulmonary function indicators highly correlated with GINA severity grading (i.e., FEV1/FVC and FEV1%pred) were significantly and negatively associated with social phobia scores. These findings indirectly support the possibility of a “dose–response relationship” between the two conditions to a certain extent.

This study further demonstrated that the two social phobia scores were associated only with leukotriene but not with other inflammatory indicators such as eosinophils, IgE, and histamine. This selective association may be explained by the fact that psychosocial stress related to social phobia is primarily linked to asthma pathology through non-allergy-specific pathways. Specifically, it appears to preferentially involve leukotriene-associated airway inflammation and smooth muscle contraction pathways, rather than IgE- and histamine-mediated allergic response pathways or eosinophil-dominated classical inflammatory pathways.

In addition, the results of BAI-related analyses further corroborate the association between general anxiety and asthma and support the notion that inflammatory responses (represented by eosinophil and leukotriene) may be involved in the underlying mechanisms linking the two conditions.

Notably, the cross-sectional design inherently limits causal interpretation of the observed association. Specifically, this design cannot clarify the chronological order of onset between social phobia and asthma, making it difficult to determine whether the two conditions exacerbate each other, whether only a one-way causal effect exists, or whether other unmeasured factors drive both outcomes. While a prospective cohort study could theoretically address this chronological limitation through long-term follow-up, it faces significant practical challenges. These include difficulty in recruiting a sufficiently large number of adolescents diagnosed with either asthma or social phobia, the need for several years of follow-up, and the high mobility of adolescents, which easily leads to loss-to-follow-up bias. In addition, repeated assessments of both asthma and social phobia during the follow-up period would inevitably increase the complexity and cost of the study. Despite these limitations, the cross-sectional design of this study still holds significant value. By using well-matched case–control samples with adjusted confounding factors, this study efficiently provides preliminary evidence of a significant association between social phobia and asthma, laying a foundation for subsequent research.

Given the observed association and its limitations in causal inference, three potential mechanisms may help explain the association between social phobia and asthma: (1) Neuro-endocrine-immune axis: anxiety induced by social phobia activates the hypothalamic–pituitary–adrenal axis and sympathetic nervous system, disrupting cortisol rhythms, impairing airway immunoregulation, and enhancing airway hyperresponsiveness through the release of norepinephrine. Conversely, physical discomfort such as chest tightness during asthma attacks may be interpreted by the central nervous system as an intense stress signal, further activating this axis, exacerbating anxiety, and forming a bidirectional regulatory cycle at the physiological level (25, 26). (2) Inflammatory pathway crosstalk: psychosocial stress from social phobia induces the release of pro-inflammatory cytokines, which “cross-activate” asthma-related inflammatory pathways. At the same time, peripheral inflammatory cytokines from persistent asthma may act on brain regions involved in emotional regulation, strengthening the perception of social fear and creating an interaction between inflammation and emotional processing (27, 28). (3) Bidirectional environmental–psychological cycle: social avoidance behaviors caused by social phobia may reduce physical activity and weaken social support, thereby compromising the standardized management of asthma. Conversely, daily functional limitations and stigma associated with asthma may further reduce adolescents' willingness to engage in social interactions, intensifying social anxiety and forming a vicious cycle at the behavioral and psychological levels (29, 30). It should be emphasized that these mechanisms are preliminary speculations based on current findings. Their validity and causal direction require further validation through empirical research.

Based on the key result of the association between Mini-SPIN scores and asthma in Table 5 (OR = 1.192), calculations using G*Power 3.1.9.7 software (*α* = 0.05, 1 − *β* = 0.95) yielded an effect size of 0.025, indicating a weak association between the two. The theoretically required minimum sample size exceeds 10,000 cases, which is substantially larger than the 674 participants enrolled in this study. This weak association aligns with existing knowledge, as both conditions involve multidimensional pathogenic mechanisms. While increasing the sample size could improve the statistical power of studies for detecting weak associations, practical constraints often make this goal unachievable. This limitation represents a typical challenge of exploratory research rather than a flaw in the design of the present study. Under such circumstances, the current study strictly controlled for confounding variables and minimized the impact of random factors. Consequently, a significant association between social phobia and asthma was still detected despite the relatively small sample size, marking the first verification of this association in adolescents. The conduct of this study confirms the feasibility of conducting small-sample exploratory research on psychosomatic comorbidity and provides a precise design basis for subsequent multicenter, large-sample cohort studies.

The asthmatic cases in this study were restricted to individuals who had experienced an acute exacerbation within the recent 3 months. This subgroup presents with more unstable disease status and distinct psychosocial stressors, which may amplify the observed association between social phobia and asthma. Four key considerations informed this design choice. First, exploring social phobia status and its short-term association with asthma after acute exacerbation aligns closely with the urgent needs of clinical practice. Second, patients with recent acute exacerbations exhibit more prominent asthmatic symptoms and clearer pulmonary function patterns, facilitating accurate asthma diagnosis and reducing misdiagnosis-related bias. Third, this selection serves as a pragmatic measure to address the large sample size requirement for verifying the weak association between asthma and social phobia. Fourth, we maximized adjustment for social phobia-related risk factors unrelated to asthma (e.g., family factors) via multivariate models to control for potential biases. Future studies may expand the sample size and include patients in remission to further validate the stability of this association.

This study still has three limitations that, while not undermining the core conclusions, highlight directions for future research. First, although major confounding factors were adjusted for, adolescent-specific psychosocial factors (e.g., school bullying experiences, parent–child relationship quality) were not included. These factors may indirectly influence asthma by exacerbating social phobia. Nevertheless, the accurate assessment of social phobia using multiple scales helped mitigate the interference of such unmeasured variables to a certain extent. Future studies should incorporate these factors for further verification. Second, samples were recruited only from adolescents in 24 communities within a single city, leading to limited geographical and ethnic representativeness and preventing direct generalization of the conclusions to other populations. However, the core pathological associations between social phobia and asthma in adolescents are likely to possess a certain degree of universality, allowing these findings to serve as a basic hypothesis for validation in diverse populations. Third, the assessment of social phobia relied solely on scales without incorporating clinical diagnoses, which may have affected the judgment of association strength. Nevertheless, the reliability of the scale-based assessment was supported by a dual-researcher consistency test, ensuring the quality of the core data. Future studies may refine in the evaluation by integrating clinical diagnoses and conducting long-term follow-up assessments.

In conclusion, this cross-sectional study confirmed that elevated social phobia scores (assessed via the Mini-SPIN and SASC) were significantly associated with an increased risk of asthma in adolescents. This association remained consistent across subgroups stratified by age at initial onset of asthma and retained a certain degree of independence after adjusting for key confounding factors. However, the cross-sectional design limits causal inference. Therefore, the specific causal relationship between social phobia and asthma (e.g., directionality or bidirectionality) awaits further verification through prospective studies. These findings highlight the need for bidirectional attention to physical and mental health in adolescents with asthma or social phobia. Clinicians may consider incorporating brief psychological assessments for asthmatic adolescents (especially those experiencing acute exacerbations) and monitoring appropriate asthma-related risks among individuals exhibiting social phobia tendencies. However, given the weak association and cross-sectional limitations, any psychological or medical interventions should be viewed as adjunctive rather than standalone approaches, and generalized application requires further validation.

## Data Availability

The raw data supporting the conclusions of this article will be made available by the authors, without undue reservation.
