# Supplementary material for: Association between social phobia and allergic asthma in adolescents
Source: Front Allergy. 2025 Dec 5;6:1698470. doi: 10.3389/falgy.2025.1698470 (PMC12714957; doi:10.3389/falgy.2025.1698470)
Supplement: Supplementary file 1 [file Table1.docx]

**Supplemental Table 1 Definitions of a series of risk factors in this study**

| **Risk factor** | **Definition** |
| --- | --- |
| **Risk Factors for**  **Asthma** |  |
| Food allergy | In the past 12 months, after consuming a certain food, at least two symptoms such as skin itching, rashes, lip swelling, vomiting, diarrhea, and wheezing occurred, and the allergy to this food was confirmed by a doctor's diagnosis or professional allergy tests (such as skin prick tests, serum specific IgE tests). |
| Inhalant allergy | In the past year, after exposure to common inhalants such as pollen, dust mites, and animal dander, respiratory allergy symptoms such as nasal itching, sneezing, runny nose, coughing, and wheezing frequently occurred. These symptoms occurred at least 3 times a year, and the relevant allergen tests (such as skin prick tests, serum specific IgE tests) were positive. |
| Industrial pollution | The residential environment is less than 1 kilometer away from factories (such as chemical, steel, and building material factories that produce pollution) in a straight line, or a pungent smell from factory emissions can be smelled for a long time (at least 3 days a week for more than 6 months). According to the air quality data released by the local environmental protection department, in the past year, the average concentration of industrial pollutants (such as sulfur dioxide, nitrogen oxides, particulate matter, etc.) in this area exceeded the national air quality grade II standard for more than 20% of the days. |
| Exhaust gas pollution | In the past year, according to the monitoring data of the environmental protection department, the annual average concentration of major pollutants in vehicle exhaust (such as carbon monoxide, hydrocarbons, nitrogen oxides, etc.) in the area where the subject lives exceeded the national air quality grade II standard. Or the subject is often exposed (at least 4 days a week) to traffic - congested roads during daily travel, and each exposure time exceeds 30 minutes. |
| Kitchen fume  pollution | In family cooking, high - fume cooking methods (such as frying and stir - frying) are used more than 5 times a week, and the kitchen ventilation equipment (such as range hoods) cannot effectively exhaust the fumes (there is obvious fume residue in the kitchen, and a strong fume smell can still be smelled after staying for 15 minutes), lasting for more than 6 months. |
| Decoration pollution | The living place has been renovated within the past year, and the decoration materials (such as paint, boards, glue, etc.) do not meet the environmental protection standards (without relevant environmental protection certification marks). The ventilation time after decoration is less than 3 months before moving in. |
| Passive smoking | In the past year, for at least 3 days a week, the subject was exposed to the smoking environment of others for more than 30 minutes a day, and the indoor smoke concentration was high (the smell of smoke can be clearly smelled, or there is obvious smoke lingering in the room). |
| Lack of exercise | Engaging in moderate - intensity or above physical activities (such as running, swimming, playing basketball, etc., with an accelerated heart rate and rapid breathing during exercise) less than 3 times a week, and each activity lasts less than 30 minutes, lasting for more than 3 months. |
| Obesity | According to the WHO growth standards for children and adolescents, calculate the body mass index (BMI), where BMI = weight (kg)÷height² (m²). If the BMI value is higher than the 95th percentile of the BMI growth curve for children and adolescents of the same age and gender, it is determined as obesity. |
| Respiratory tract  infection | In the past year, the number of medical consultations due to respiratory diseases such as colds, influenza, and pneumonia exceeded 3 times, or the cumulative duration of symptoms such as persistent coughing, fever (body temperature ≥ 38°C), and rapid breathing caused by respiratory tract infections lasted for more than 10 days. |
| Family history  of asthma | At least one of the first - degree relatives (parents, siblings) has been diagnosed with asthma by a doctor. |
| **Risk Factors for**  **Social Phobia** |  |
| Relative's death | In the past 5 years, a close immediate family member (parents, grandparents, siblings) or an important collateral family member (such as an uncle, aunt, etc. who has taken care of the subject for a long time) passed away. |
| Parental divorce | The subject's parents went through the divorce process during the subject's age from 10 to 19. |
| Parent unemployed | One or both of the subject's parents were unemployed in the past year. |
| Over - indulgence | The parents almost unconditionally meet the subject's needs. The subject does no housework and the parents always solve problems for them immediately, with this parenting style lasting over 2 years. |
| Lack of parental care | The daily effective communication time between the parents and the subject (focused communication, discussing topics such as study, life, and hobbies) is less than 30 minutes, and the number of parent - child activities such as outdoor activities and shared meals per week is less than 3 times, lasting for more than 6 months. |
| Poor academics | In the past academic year, the average score of major subjects (Chinese, mathematics, English, etc.) was more than 20 points lower than the class average, or the subject's test score ranked in the bottom 20% of the class in school - organized exams. |
| School relationship  problems | In school, the number of conflicts with classmates or teachers (such as arguments, fights, being isolated by classmates, etc.) exceeds 2 times a month, or due to interpersonal relationship problems, the number of days when the subject cannot participate in normal school activities (such as physical education classes, recess activities, club activities, etc.) accumulates to more than 15 days, lasting for more than 3 months. |
| Introverted &  sensitive | In social situations, the subject often shows shy and withdrawn behaviors. When facing strangers or new environments, the subject is prone to nervousness, blushing, and an accelerated heart rate. This situation occurs in more than 70% of social situations and has lasted for more than 1 year. It is further confirmed by personality tests (such as a score higher than the norm in the introversion dimension of the Eysenck Personality Questionnaire for Adolescents). |
| Sick body | In the past year, due to physical diseases (such as chronic pain, chronic diseases, etc.), the number of days when the subject could not participate in normal school activities (such as physical education classes, recess activities, etc.) accumulated to more than 30 days. |
| Insufficient sleep | According to the expert consensus on adolescent sleep health, adolescents aged 10 - 19 should ensure 8 - 10 hours of sleep per day. If the subject's average daily sleep time in the past 3 months is less than 7 hours. |
| Addiction to  the Internet | According to the diagnostic criteria for Internet addiction, the subject's weekly Internet - using time exceeds 20 hours, and the Internet is mainly used for entertainment (such as playing games, watching videos, etc.) rather than learning, lasting for more than 3 months. |
| Lack of hobbies | In the past year, except for school - arranged courses and homework, the subject did not have any hobbies that were persisted in for a long time (more than 3 months) (such as painting, music, sports, etc.). |
| Media influence | The subject spends more than 3 hours a day watching TV, online videos (such as short videos, TV dramas, movies, etc.) or playing video games, lasting for more than 6 months. |
